# Supplementary material for: Efficacy of Chondroprotective Food Supplements Based on Collagen Hydrolysate and Compounds Isolated from Marine Organisms
Source: Mar Drugs. 2021 Sep 26;19(10):542. doi: 10.3390/md19100542 (PMC8541357; doi:10.3390/md19100542)
Supplement: Supplementary file 1 [file marinedrugs-19-00542-s001.zip › marinedrugs-1309586-supplementary.pdf]

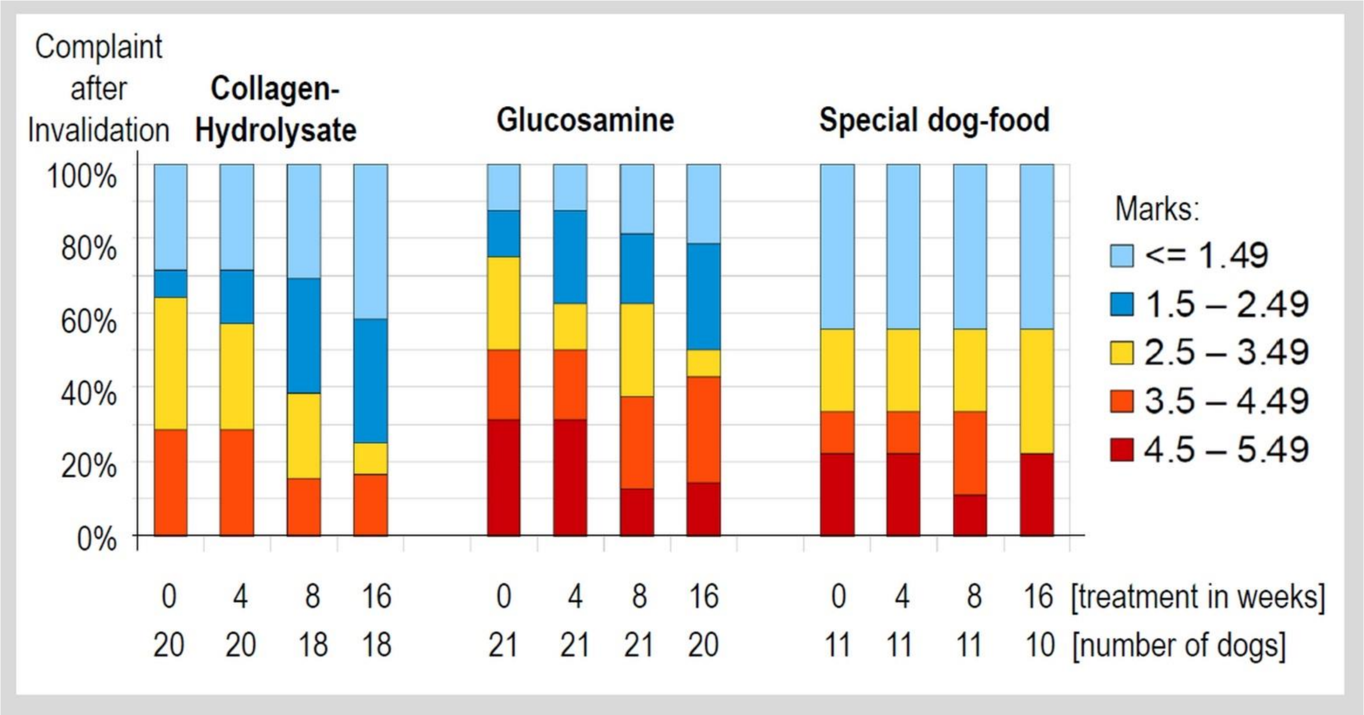

Figure S1. Assessment of Complaints after invalidation/ burden. The distribution of grades is shown differentially by colour each week at 4 week intervals during the sixteen week study period. The complaints of all dogs under study after prolonged exposure were rated with an average mark of 2.92 at the beginning of the study period. In the collagen group the average mark was 2.64, in the glucosamine group it was 3.38 and in the joint diet group it was 2.16. At the end of the study period, the average mark of all dogs was rated 2.42. In the collagen group it was 1.93, in the glucosamine group it was 2.8 and in the joint diet group it was 2.46. As the diagram shows, the dogs in the glucosamine group tend to have more severe symptoms after prolonged exercise. (Descriptive assessment of the data)

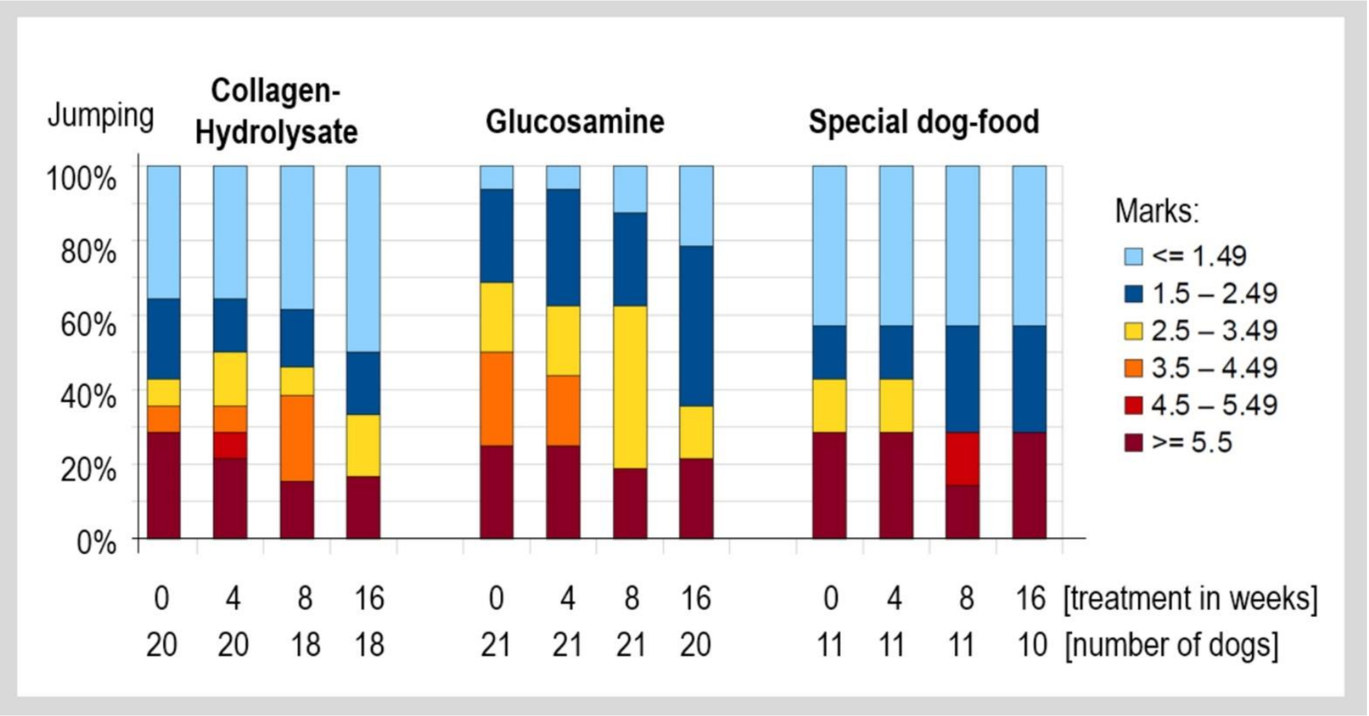

Figure S2. **Jumping study of the dog patients.** The distribution of jumping ability is shown differentially by colour each week at 4 week intervals during the sixteen week study period. The average mark for jumping was rated with a mark of 3.24 at the beginning of the study period. In the collagen group the average mark was 3.0, in the glucosamine group 3.63 and in the joint diet group 2.86. At the end of the study period the average mark of was 2.6, in the collagen group 2.34, in the glucosamine group 2.79 and in the joint diet group 2.68. Figure 2S shows that the dogs in the glucosamine group tended to have more discomfort when jumping. A conspicuous smaller number of animals jumped completely symptom-free in the joint diet group, the number of animals that jumped without discomfort did not increase.

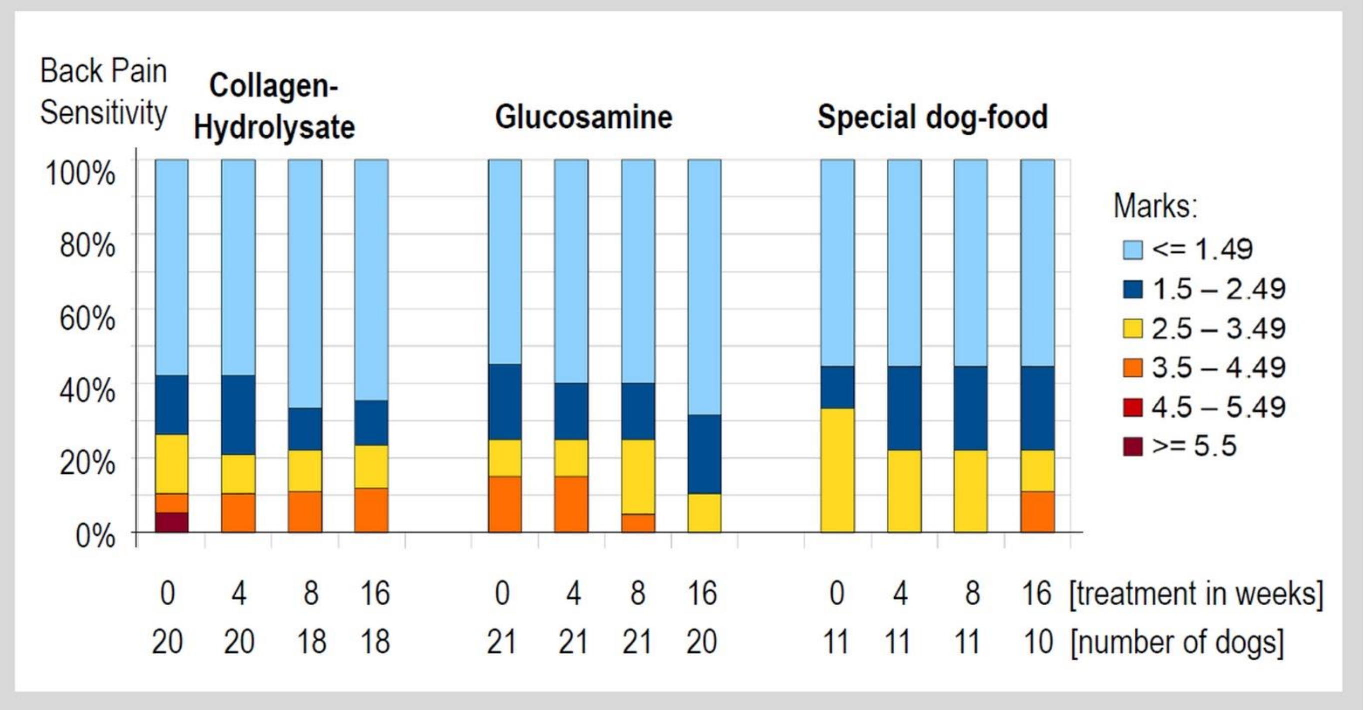

Figure S3. **Back pain touch sensitivity of the dog patients.** The distribution of back pain sensitivity is shown differentially by colour each week at 4 week intervals during the sixteen week study period The touch sensitivity of the back was rated with an average mark of 1.85 at the beginning of the study period. In the collagen group the average was 1.89, in the glucosamine group 1.85 and in the joint diet group 1.78. At the end of the study period, the average was 1.59. In the collagen group at 1.69, in the glucosamine group at 1.48 and in the joint diet group at 1.63. groups are characterized by a certain homogeneity.

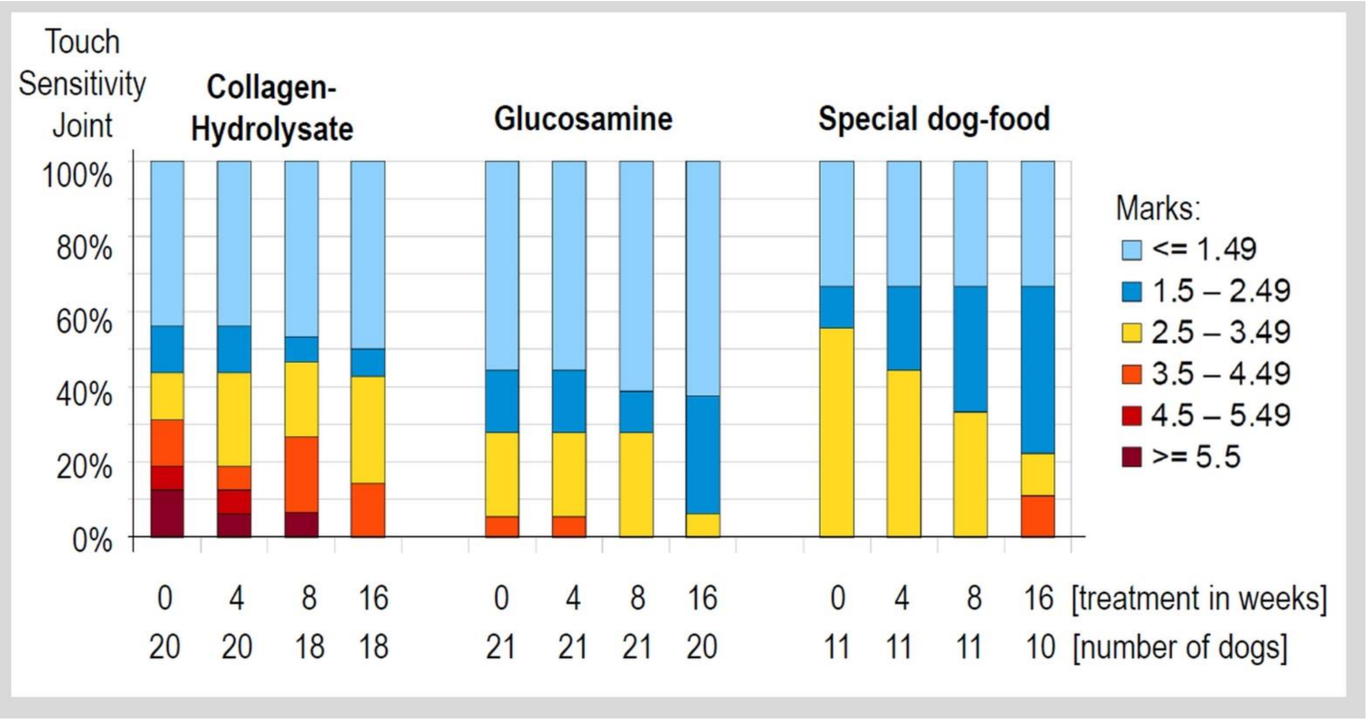

Figure S4. **Joint pain sensitivity of the dog patients.** The distribution of grades is shown differentially by colour each week at 4 week intervals during the sixteen week study period. The touch sensitivity of the affected joint was rated with an average mark of 2.19 at the beginning of the study period. In the collagen group the average was 2.63, in the glucosamine group 1.78 and in the joint diet group 2.22. At the end of the study period the average was 1.97. In the collagen group it was 2.4, in the glucosamine group it was 1.63 and in the joint diet group it was 1.94. A noticeable decrease in the sensitivity to touch was found in the collagen and glucosamine groups during the study period. Overall, by description of the data, the difference in joint pain sensitivity between the groups was not conspicuous at any time of the study. However, a trend is apparent with touch sensitivity decreasing for all three groups over the course of the study (grade 1.5-2.49).

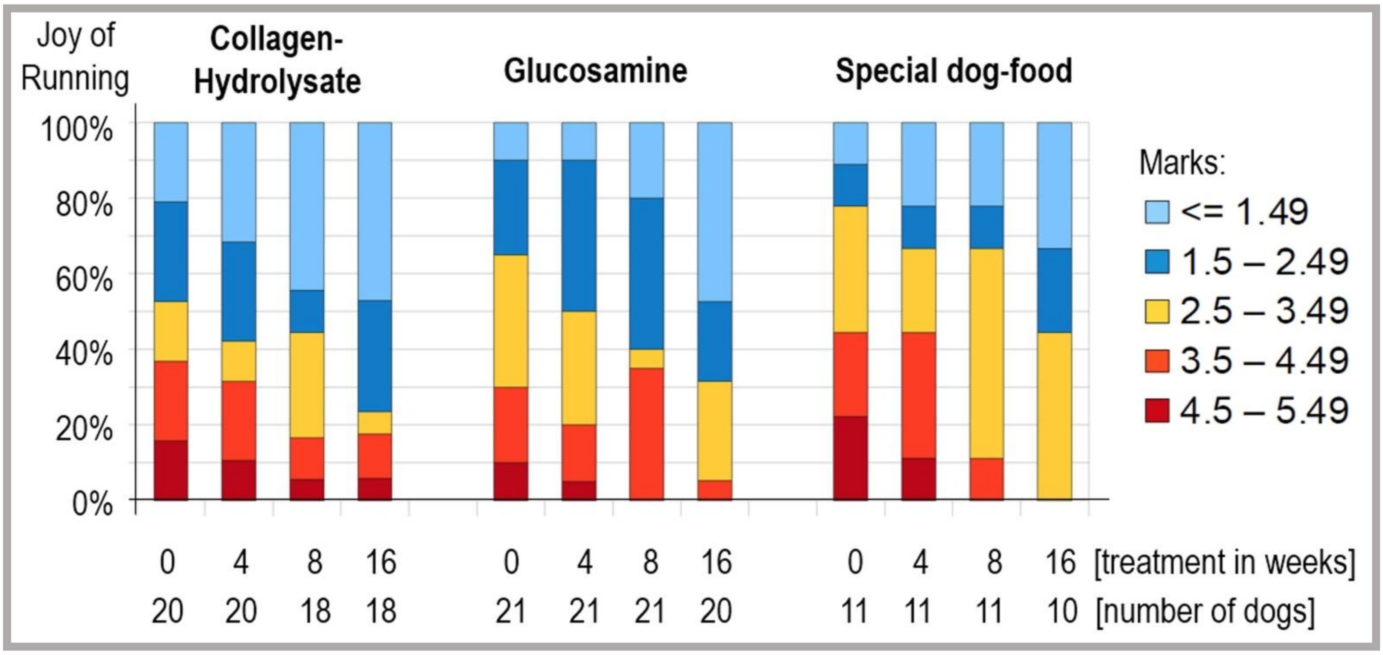

Figure S5. Joy of running marks of the dog patients as revealed by the patient-holders. The dogs' joy of running was rated at an average of 2.96 at the beginning of the study period. In the collagen group the average was 2.84, in the glucosamine group 2.93 and in the joint diet group 3.28. At the end of the study period, the average was 2.03 in the collagen group, in the glucosamine group at 1.99 and in the joint diet group at 2.12. The frequency distribution in the three groups with regard to the grades awarded is shown at the right side.

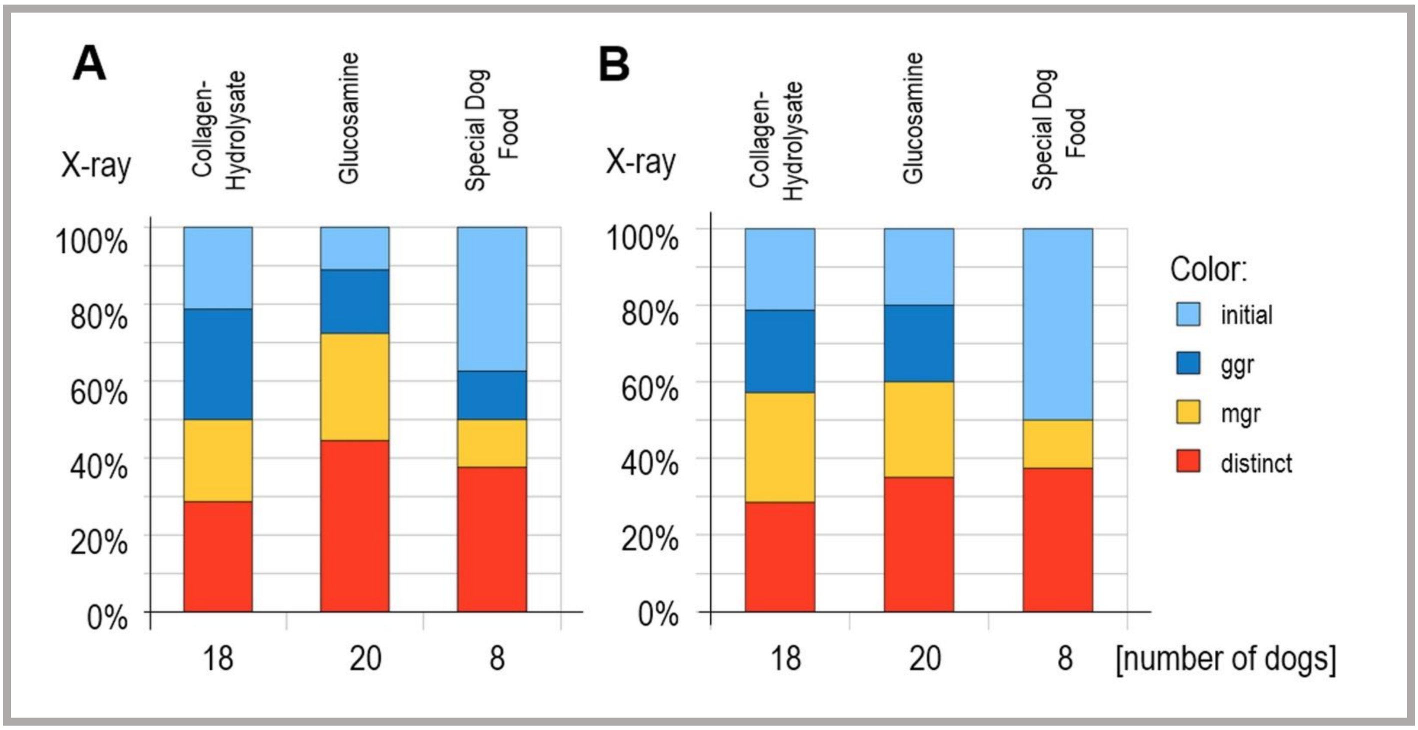

Figure S6. X-ray data of the left and right femoral joints (**A** left; **B** right) obtained from each dog at the beginning of the study. There was no no statistical deviation detectable between the right and left hip joint. This is also the case when comparing the left and the right knee joint (p-value left knee joint: 0.12; right knee joint: 0.13; left hip joint: 0.11; right hip joint: 0.15).

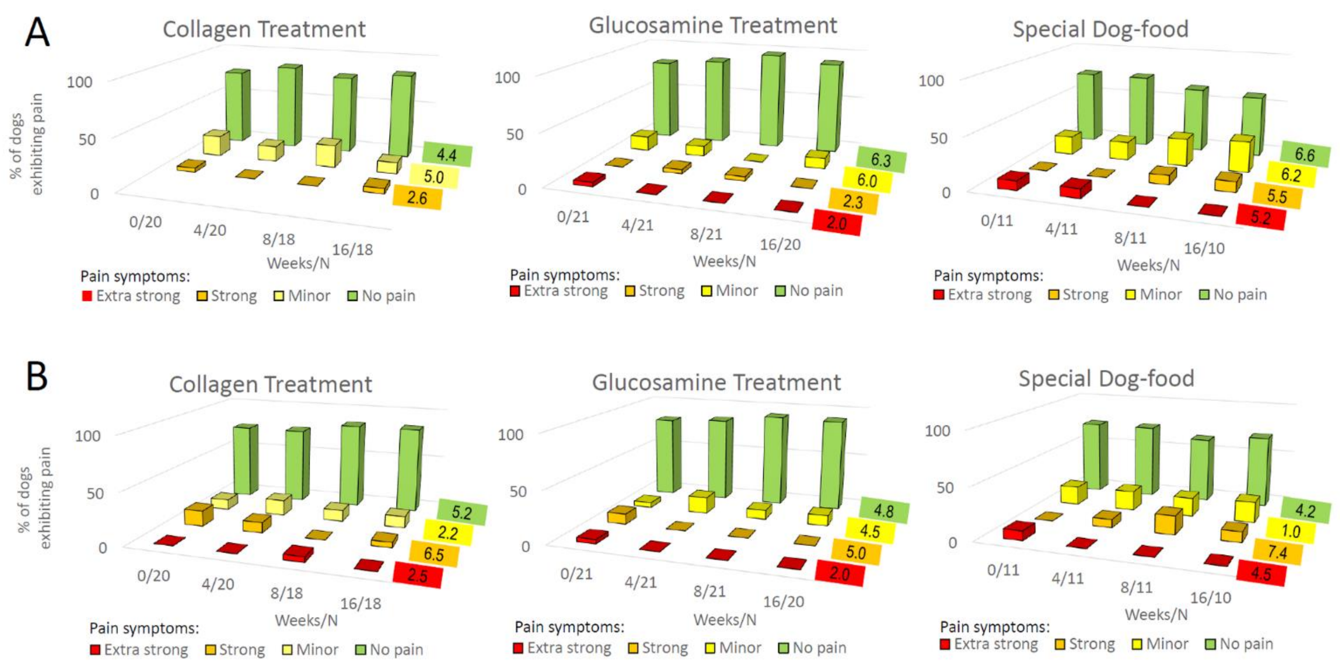

Figure S7. Canine dog patients with beginning OA symptoms were examined at the different time frames, i.e. beginning (0) and after 4, 8 and 16 weeks of treatment concerning pain in their A) left or B) right femoral joint. Pain symptoms during palpation were examined for all dogs in the three groups according to a clinical standard protocol. Four scores are given: no pain reaction (green), minor (yellow), strong (orange) and extra strong pain reaction (red). The pain distribution of the dog patients is shown in percent for the number of dogs N within the groups examined with standard deviation (SD) values shown in the column on the right.

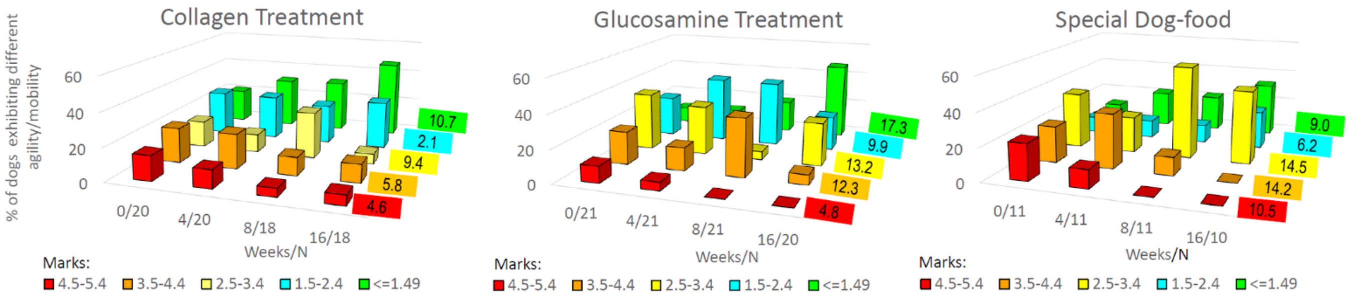

Figure S8. Agility and mobility of dogs with beginning OA symptoms. Marks/color coding: green - no problems, blue - minor problems, yellow - moderate problems, orange - large problems, red - extremely large problems. The marks were given by the animal-holders at different time-points (see Figure 1 for details) for the groups of dogs as indicated. These marks correlate with QOL. Standard deviation (SD) values are shown in the column on the right.

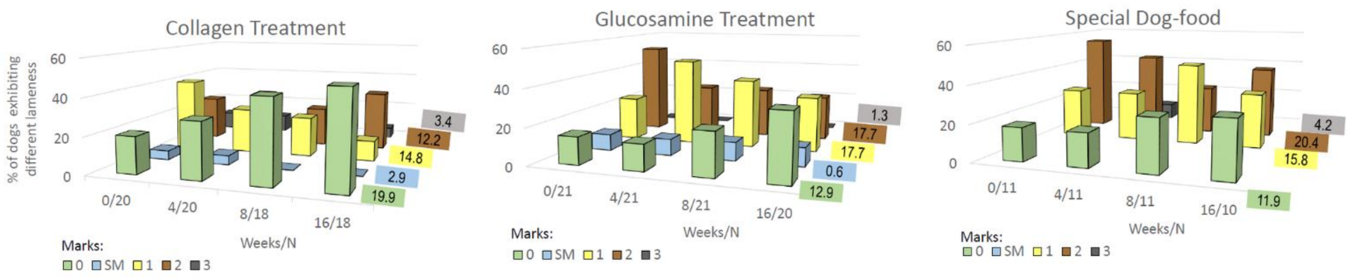

Figure S9. Lameness of dogs with beginning OA symptoms. The grades were given by a veterinarian not further involved in the study but viewing and evaluating the video footage at both the beginning and end of the therapy. Grades/color coding: green - no problems (0), blue - minor problems (marked SM - stiff movement), yellow - moderate problems (1), brown - large problems (2), grey - extremely large problems (3). Standard deviation (SD) values are shown in the column on the right.
